# Carbohydrates—Key Players in Tobacco Aroma Formation and Quality Determination

**DOI:** 10.3390/molecules25071734

**Published:** 2020-04-09

**Authors:** Marija Banožić, Stela Jokić, Đurđica Ačkar, Marijana Blažić, Drago Šubarić

**Affiliations:** 1 Faculty of Food Technology Osijek, Josip Juraj Strossmayer University of Osijek, Franje Kuhača 20, 31000 Osijek, Croatia; marija.banozic@ptfos.hr (M.B.); dackar@ptfos.hr (Đ.A.); dsubaric@ptfos.hr (D.Š.); 2 Karlovac University of Applied Sciences, Josip Juraj Strossmayer Square 9, 47000 Karlovac, Croatia; marijana.blazic@vuka.hr

**Keywords:** tobacco, carbohydrates, aroma profile, processing condition

## Abstract

Carbohydrates are important compounds in natural products where they primarily serve as a source of energy, but they have important secondary roles as precursors of aroma or bioactive compounds. They are present in fresh and dried (cured) tobacco leaves as well. The sugar content of tobacco depends on the tobacco variety, harvesting, and primarily on the curing conditions (temperature, time and moisture). If the process of curing employs high temperatures (flue-curing and sun-curing), final sugar content is high. In contrast, when air curing has a lower temperature, at the end of the process, sugar level is low. Beside simple sugars, other carbohydrates reported in tobacco are oligosaccharides, cellulose, starch, and pectin. Degradation of polysaccharides results in a higher yield of simple sugars, but at the same time reduces sugars oxidization and transfer into carbon dioxide and water. Loss of sugar producers will compensate with added sugars, to cover undesirable aroma properties and achieve a better, pleasant taste during smoking. However, tobacco carbohydrates can be precursors for many harmful compounds, including formaldehyde and 5-hydroxymethylfurfural. Keeping in mind that added sugars in tobacco production are unavoidable, it is important to understand all changes in carbohydrates from harvesting to consuming in order to achieve better product properties and avoid the formation of harmful compounds. This review summarizes current knowledge about tobacco carbohydrates, including changes during processing with special focus on carbohydrates as precursors of harmful compounds during smoking.

## 1. Introduction

Tobacco (*Nicotiana tabacum* L.) is the most widely cultivated non-food crop in the world. Although not consumed as a food, it is partially consumed by digestive system (mouth) and its sensory properties such as aroma, flavor and odor are the most important attribute to define consumer acceptance of tobacco and tobacco products. Besides consuming by smoking, a small part of total tobacco production (less than 10%) is for consummation by chewing and sniffing [1,2]. Sensory properties of products may help to determine whether a product will be preferred over another. To control sensory qualities of these types of products, it also appears necessary to understand the mechanisms involved in the retention and releasing of aroma compounds [3]. Different complex chemical reactions are involved in tobacco aroma formation, including degradation of carbohydrates, degradation of chlorogenic acid, degradation of proteins, Maillard reaction, Strecker degradation and caramelization reactions, and many others. For centuries, carbohydrates were considered as primary compounds with high nutritional value. Today it is well known that carbohydrates have both digestive and psychological role. The term carbohydrates is generally considered as neutral compounds of carbon, hydrogen and oxygen and is used to refer to sugars, oligosaccharides, and polysaccharides combined. The term sugars has been applied to simple carbohydrates (monosaccharides and disaccharides). The more complex carbohydrates containing 3 to 10 sugars refers to term oligosaccharides, and the term polysaccharides was used for even more complex carbohydrates (starch, cellulose and pectin). Some of them are digestible in whole (sugars) and some of them represent totally indigestible nutrients (indigestible fibers) [4]. Tobacco is a complex mixture that consists of 6–15% cellulose, 10–15% pectin, roughly 2% lignin, variable contents of sugars, and a variety of other components (Table 1). The exact composition depends on the tobacco part or variety and the growing conditions [5]. Tobacco variety, growing conditions, chemical composition, and many parameters during processing have an influence on carbohydrate contents. Thus, uniform quality of final products is the main goal, and a critical point in tobacco production. Better understanding of tobacco carbohydrates and their impact on tobacco flavor and aroma as well as on the formation of undesirable compounds could be crucial. Present knowledge about tobacco carbohydrates and their impact on aroma profile originates from recently published articles [6,7,8,9,10,11,12,13]. Together with nitrogen compounds, tobacco carbohydrates are the most important aroma precursors [6,14]. Since they have low vapor pressure, they are sensitive to high temperatures and oxidation processes [15]. Besides carbohydrates, other aroma compounds are derived from carotenoid degradation products [8].

The overall objective of this review is to evaluate tobacco carbohydrates as precursors of desirable and undesirable tobacco aroma compounds on previously published studies bases. This review summarizes tobacco changes during processing, from harvesting to the end of curing and provides better understanding of the role of added sugars in tobacco production. Particular emphasis was placed on both sides of carbohydrates, as desirable aroma compounds precursors, and harmful compound precursors as well. There is a lack of published papers looking into carbohydrate changes during tobacco processing. To the best of our knowledge, this is the first systematic review considering changes of all carbohydrates (not only changes in simple sugars content), and different roles of carbohydrates (as naturally present compounds, additives, precursors of aroma and precursors of harmful compounds) in tobacco.

## 2. Tobacco Carbohydrates

Carbohydrates are naturally present in tobacco but some extra amounts are frequently added during processing. Content of complex carbohydrates decreases during processing, but sugar content is mostly constant, because loss of sugars during processing is compensated with starch decomposition and addition of sugars.

### 2.1. Tobacco Sugars

Most abundant naturally present sugars in dried tobacco leaves are glucose, fructose and sucrose [16]. Noteworthy differences in sugar content were found between tobacco varieties, i.e., *Virginia* has high level of sugar (8–30%) while *Burley* is characterized with low content of sugars (1–2%) [17]. On the other hand, *Oriental* tobaccos possess significant amounts of sugars (10–20%) and lower amounts of proteins, probably because they are usually planted on low nutrient soils with limited amounts of nitrogen and water, and cured directly on the sun [6,18]. As a consequence, acetate accumulates in the Krebs cycle, resulting in biosynthesis of aromatic compounds—terpenoids. These types of terpenoids are usually synthesized from mevalonic acid, carbohydrates, acids, and resins, which contain nitrogen [19].

High-level sugar tobaccos (8–30%) are usually preferred by tobacco producers and consumers, because tobacco product preferability is proportional to the sugar level [20]. There are two main types of tobacco blends in cigarette production, American and *Virginia* blends. *Virginia* blends are composed mainly by *Virginia* tobacco and other similar bright tobaccos while American blends are composed of significant amount of *Burleys* tobacco and small amount of *Oriental* tobaccos [6]. Roasted *Burley* tobacco constitutes about 30% of American cigarette blends. Roasting includes high temperature treatment (350 °C) of *Burley* tobacco and as a result of catabolic breakdown, total sugar content is very low (around 1.2%). Wu and Wu [21] reported different changes in *Burley* tobacco during roasting, including changes in reducing sugars content, total water-soluble sugars content, total nitrogen content, total alkaloid contents, and total volatile compound contents. Roasting is an important step, not only for generating aroma compounds, but also for removing amines, reducing irritation, modifying the pH, and generally improving the tobacco quality [22]. Since American blends are the most widely used blends for cigarette manufacturing and addition of sugars is unavoidable, it is important to understand potential influence of naturally present and added sugars on tobacco quality and toxicity. Determination of carbohydrate content in tobacco leaves can be conducted using segmented flow analysis, gas chromatography [23], liquid chromatography [24], and ion chromatography [25].

Sometimes tobacco producers add carbohydrates, primarily sugars into tobacco blend to compensate for loss of sugars during the process of curing [6]. They are approved as additives in tobacco products in amounts up to 10% in UK [26], while European Union did not define the maximum amount of added sugars. Generally for American blends, sugars are added in amount up to 5%, which results in lower total sugar content than in *Virginia* blends (less than 11%). Most frequently used tobacco sugar additives are sucrose and invert sugars [6]. Some of the producers add glycerin or cellulose as humectant in tobacco blends [16]. Types of tobacco that are not used for smoking (chewing tobaccos) are usually rich in added sugar solutions to achieve sweet taste. Using subjective sensory evaluation in the research of Baker et al. [15], panels described tobacco taste with added sugar as sweet, precisely pleasant, mild, and non-harsh. Carbohydrate additives and other additives are added during a special process named “saucing”. Added sauce is a mixture of flavorings, citrus and fruit extracts, and sugars; sometimes sugars even include corn syrup, molasses or honey [13]. European Union directive from 2014 obliged tobacco producers to report all tobacco ingredients, including added sugars [27]. However, the mentioned directive did not regulate maximum amount of added carbohydrates but only suggested that producers should reduce usage of additives. Moreover, they recommend that sugar additives should be added in amounts that only replace sugars lost during processing and not to achieve a better (sweet) taste of tobacco. Roemer et al. [6] reported that 0.5% of the added sugar content is transferred unchanged into smoke. Following that claim, the maximum daily dose of sugar intake from smoking would be around 0.008 g per day.

### 2.2. Tobacco Oligosaccharides

Oligosaccharides are a less-represented group of carbohydrates in tobacco. In tobacco seeds, the presence of planteose is reported, in green leaf raffinose and stachyose, and in dry tobacco leaves erlose and theanderose are present [24]. Malto-oligosaccharides are mostly products of starch degradation, but later in the process they decompose and contribute to Maillard reactions [28]. Nagai et al. [24] investigated oligosaccharides (fructo-oligosaccharides and malto-oligosaccharides) in tobacco fresh and dried leaves. Results showed that fructo-oligosaccharides were completely absent in fresh leaves and their content increased during processing due to enzymatic reactions, while malto-oligosaccharides degraded during processing due to the influence of heat. Increasing contents of fructo-oligosaccharides could be explained by the degradation of carbohydrates, because tobacco is a non-fructan plant [29].

### 2.3. Tobacco Polysaccharides

#### 2.3.1. Starch

Starch is important primary metabolite in tobacco, accumulated during growing and decomposed during processing. At the end of processing, almost all starch is converted into water-soluble carbohydrates, and subsequently into aroma compounds. Although starch is necessary for aroma formation, its presence in tobacco products can cause bitterness and flavor changes [30]. Moreover, it can affect changes in color and odor of cured tobacco [11]. Therefore, complete degradation of starch is one of the important steps in tobacco processing [30]. Degradation of starch is one of the most important changes during leaf processing and will be discussed in more details separately.

#### 2.3.2. Pectins

Along with cellulose and hemicellulose, pectins are among the major compounds of tobacco cell walls, mainly composed of D-galacturonic acid, followed by D-galactose and L-arabinose [31,32]. They are widely used because of their gelling and thickening properties. Recent research by Zhang et al. [33] showed that tobacco agricultural waste-stem contains between 11.27% and 8.88% of pectins and could be a good source of pectin. Pectin content is important for tobacco quality determination. With increasing pectin content, tobacco blend becomes soft and sensitive at high air humidity, or rigid and fragile at low air humidity [34]. However, pectin is considered as an undesirable compound in tobacco product because of poor smoking characteristics [35]. Even though it is an undesirable compound in leaf processing, pectin has an important role in the production of reconstituted tobacco. Reconstituted tobacco are sheet-like papers, composed of tobacco stem and leaf particles. Sometimes it is added into tobacco blends, especially for low-price cigarettes. There are few commercial processes for reconstituted tobacco production. One of the most commonly used processes includes treatment of tobacco waste material solution with diammonium phosphate in order to dissolve tobacco pectins. During that process, under heat influence, part of the ammonia content is volatilized and pectin acts as binding material and keeps reconstituted tobacco sheets together. In addition to adhesive property, in this case, pectin plays an important role for smoking quality because the content of some aroma compounds (pyrazines) increases [19,36].

#### 2.3.3. Cellulose

Primary and secondary cell walls of tobacco are composed of cellulose, hemicellulose and pectin [36]. Content of cellulose can be up to 40% for stem parts, up to 15% for midrib and up to 15% for leaf lamina [19]. In tobacco plant metabolism, cellulose is not a reserve material but mechanical strengthener and protector from outside factors. Earlier, cellulose was considered as a neutral compound, which did not affect tobacco aroma [37], but today it is well known that high cellulose content contributes to negative aroma properties. Since pyrolysis of cellulose delivers undesirable taste, using stems in cigarette production is avoided [19,38]. The volatile fraction of tobacco contains furancarboxaldehyde and its derivatives and it is assumed that they originate from cellulose in lamina cells [39]. Torikaiu et al. [40] also proved an increase of formaldehyde yield and decrease of aromatic amines with increasing content of cellulose in tobacco leaves, while Sanders et al. [41] reported that pyrolysis of tobacco cellulose had resulted in a high level of low molecular compounds, such as ketones and aldehydes.

## 3. Changes during Processing of Tobacco Leaves

**Figure 1:**
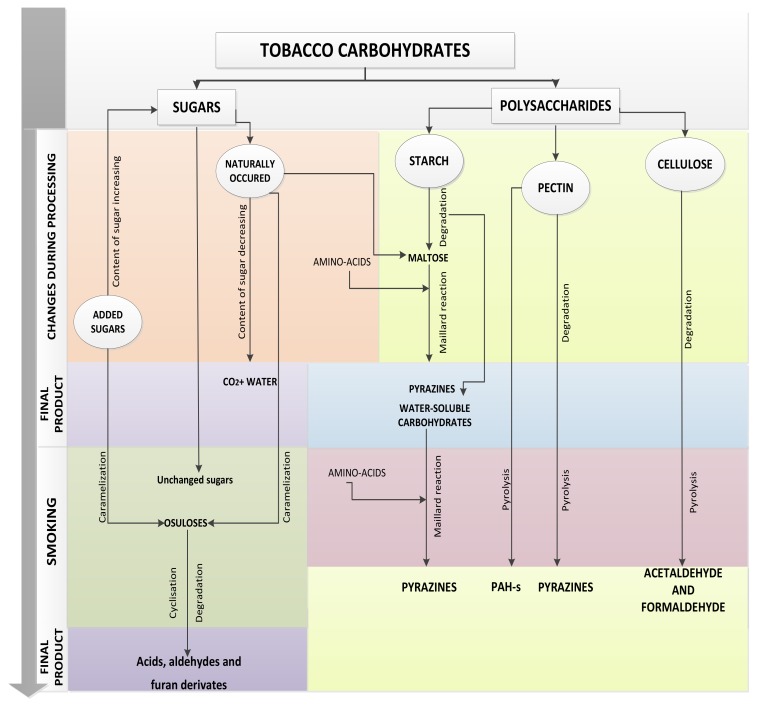
Changes in carbohydrates during tobacco processing and smoking.

In order to achieve favorable physical and chemical properties, tobacco leaves are subjected to different processes, injury, and heat and osmotic stresses [42]. During the process of curing, which includes aging, yellowing, browning, drying, and fermentation, different chemical changes occur in controlled conditions of temperature, moisture content and ventilation. The first phase of curing is yellowing, marked with many chemical changes and changes in color. During this phase, chlorophyll starts to degrade and the green color of leaf is changed into the characteristic yellow color of tobacco. Yellowing is followed by 4-day-long drying on higher temperatures for *Virginia* blends (around 55 °C). In that stage, enzymatic processes are stopped but it does not affect degradation of starch [6]. On the other hand, *Burley’s* yellowing phase is prolonged for a few weeks at relatively low temperatures (up to 40 °C), where almost all sugar is decomposed. Throughout the fermentation process, high initial moisture content is decreased, heat is generated and loss of weight occurs. The fermentation process requires precise selection of temperature (maximum 60 °C) and moisture (above 10%) conditions. For example, at a moisture content between 10% and 27%, changes in tobacco are conditioned primarily by enzymatic reaction; above 27% moisture, microbiologic reactions are dominant, and at moisture content below 10%, all enzymatic reactions stop [17]. Total carbohydrate content remains almost the same during processing, because starch content decreases while water-soluble sugar content increases (Figure 1). Sweet, roast smell appears as a result of curing [42]. On the other hand, aging is a mild fermentation process. Major reactions that take place during the process of aging are Maillard reactions between reducing sugars and amino compounds, which result in CO_2_ and melanoidins [13]. The slow process of curing at low temperatures (up to 40 °C) lasts longer and enables the enzyme system in the leaves to remain active. As a result, dry leaves have very low sugar content [6]. The process of curing is highly dependent on tobacco variety and sugar contents in fresh leaves, but it can be improved by adjusting variables such as temperature, relative humidity, air velocity, and time [43].

### 3.1. Changes in Content of Reducing Sugars

Generally, the content of reducing sugars increases during the process of yellowing. After the yellowing phase, the content of reducing sugars stagnates for some time, and then starts to decrease again. At the end of the yellowing process, reducing sugars are oxidized to the carbon dioxide and water. The final content of reducing sugars in dry leaves is highly dependent on the curing process. If the process of curing employs high temperatures (flue-curing and sun-curing) where temperature for leaf drying goes up to 60 °C, final sugar content is high. In contrast, when air curing employs lower temperature (from room temperature to 45 °C), at the end of curing, the sugar level is low. In flue-cured leaves, the content of reducing sugars is up to 22% of dry weight [43]. Previous researchers reported total amount of sugars in tobacco leaves to be up to 30%, with 22% of them representing reducing sugars [44]. Opposite to other carbohydrates (starch, cellulose, pectin), the presence of reducing sugar has a positive influence on tobacco-smoking properties, improving flavor and aroma [45].

### 3.2. Degradation of Starch

Degradation of starch starts in the first phase of curing and is predominantly caused by amylolytic enzymes. Those types of enzymes are very sensitive to temperature and moisture content. Temperatures above 70 °C and low humidity can completely stop their activity in tobacco leaves [30]. In the research of Abubakar et al. [43], most of the starch degraded within the first 80 h of the yellowing phase. Yamaguchi et al. [42] proved starch degradation in tobacco leaves during the curing process to be predominantly caused by α-amylase. Other reported enzymes involved in starch degradation are glucan water dikinase and β-amylase, but they are more important during growing, and less during leaf processing. Under normal growing conditions, starch degrades as follows. First, it is phosphorylated by glucan water dikinase, and then the phosphorylated soluble starch degradation is catalyzed by β-amylase and isoamylase, releasing considerable amounts of maltose [42]. At the same time, proteins are fragmented releasing amino acids, which will be lately contribute in Maillard reaction with sugars [6].

### 3.3. Maillard Reactions

Maillard reactions are a complex system of non-enzymatic browning reactions that have been gaining more and more attention during the past two decades. It is well known that Maillard reactions generate aroma and flavor compounds during thermal processing in the food and plant industry. Those types of processes include transformation of aroma and color precursors (sugars and amino acids) into aroma and color compounds [8]. Examples for such process are roasted meat, baked bread, roasted cocoa, and nuts [46]. Moreover, many specific beverages such as coffee, beer, whisky, and tea owe their aroma properties to the Maillard reaction. Volatile compounds derived from the Maillard reaction are generally present at trace levels, but they contribute to the characteristic flavor of a food or beverages. Cerny et al. [46] outlined that odorant systems that contain sulfur compounds have especially low yield of aroma compounds.

Maillard reactions have received much attention over the last two decades. Despite many published papers, factors influencing the formation of aroma compounds from carbohydrate/amino compounds reaction mixtures are not entirely clear [47]. Those types of reactions are highly dependent on moisture and temperature. Zhu et al. [34] concluded that low moisture content and increase of temperature during the stage of curing undoubtedly lead to the termination of the Maillard reactions. Mitsui et al. [9] found aspartic acid, proline, malic acid, and sugars (fructose, glucose, and sucrose) as compounds that contribute to Maillard reactions in tobacco. Maillard reaction precursors are present in relatively high concentrations, sugars in amounts up to 20% in fresh leaves, and even up to 30% in flue-cured tobacco, and amino acids in amounts up to 1% [48]. Maillard reactions are the main process during tobacco aging resulting in flavor compounds, melanoidins and carbon dioxide [13]. Fructose amino acids are occurring as intermediates of this reaction and they are results of the Amadori rearrangement in cured or stored tobaccos [13]. Several authors determined Amadori compounds in tobacco leaves [49] and products [48,50]. Besides curing, Maillard reactions are occurring during smoking of tobacco [6] (Figure 1).

As already mentioned, tobacco aroma profile originates from two main processes, degradation of carotenoids and Maillard reactions. Degradation of carotenoids mainly results in floral aroma compounds such as damascenone and megastigmatrienone. Maillard reactions result in different aroma profiles [30] characterized with high levels of heterocyclic compounds, mostly pyrazines [6]. Zhu et al. [8] reported furfuralcohol, furfural, 4-cyclopentene-1,4-dione, 2-methyltetrahydrofuran-3-one, 2-acetyl, and 2-acetylpyrrole as the main volatile compounds resulting from Maillard reactions. Those compounds give tobacco products a characteristic woody, caramel and baking flavor [30].

### 3.4. Caramelization

Caramelization reactions are non-enzymatic browning reactions. Unlike Maillard reactions, during caramelization, sugars do not react with amines [51]. Caramelization reactions include a complex series of different processes including oxidation, dehydration, isomerization, and polymerization [6]. During the caramelization process, sugars are decomposed into organic acids and aldehydes [20]. Dehydration generates osuloses, which can cyclise to some furan derivatives. Osuloses can also break down into acids and aldehydes [20,52]. The main degradation product of caramelization reactions is 5-HMF. As a result of caramelization process, changes in aroma profile and color occur.

## 4. Changes during Smoking

During smoking, temperature reaches between 700 °C and 900 °C and almost all carbohydrates are decomposed (pyrolysed). Products of pyrolysis of carbohydrates depend mostly on applied temperatures [5]. At temperatures up to 200 °C, all reducing sugars are decomposed. Temperatures between 200 and 250 °C are followed by the decomposition of pectin and hemicellulose [36] and at 250 °C starts degradation of pectin [35]. At temperatures above 250 °C, cellulose starts to degrade [36]. Decomposition (pyrolysis) of carbohydrates leads to an increasing level of aldehydes such as acetaldehyde and formaldehyde [53]. Formaldehyde is predominately formed by the hydroxymethyl group of sugars [6,35]. Along with pyrolysis, another process can occur, and that is pyrosynthesis. Pyrosynthesis is a process occurring under pyrolysis of tobacco where different high molecular weight compounds (larger than precursor) are formed. Combustion is another process occurring during pyrolysis, but with the presence of oxygen [6].

### Carbohydrates as Precursors of Harmful Compounds

**Figure 2:**
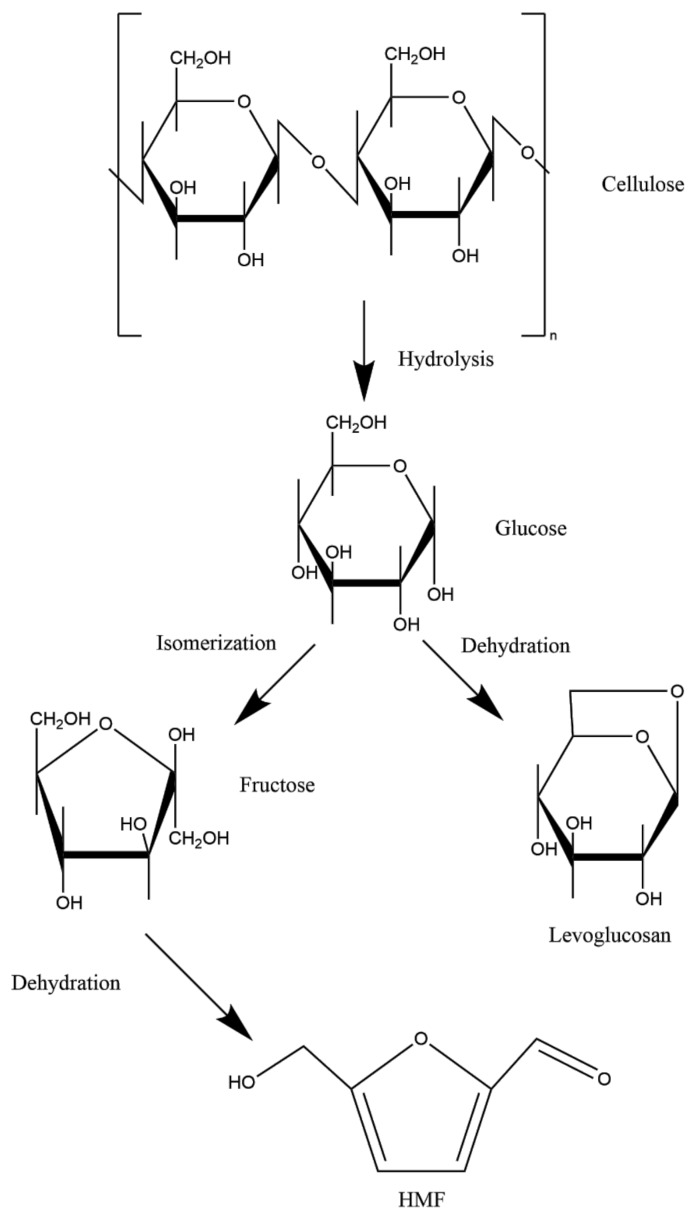
Mechanism of tobacco cellulose degradation under influence of high temperature and formation of HMF and levoglucosan (based on literature [53,54,55]).

High content of carbohydrates in tobacco leaves can lead to the formation of toxic compounds such as acetone, acrolein, 2-furfural, and formaldehyde in smoke. Some of them are carcinogenic or can raise tobacco-addictive effects [23]. Reported increase of toxic compounds in high-sugar tobaccos is up to 150% in comparison to low-sugar tobaccos [20]. Figure 2 shows a cellulose degradation pathway where under the influence of high temperature (above 250 °C), the formation of HMF and levoglucosan occurs.

Aldehyde, such as acetaldehyde and formaldehyde, are tobacco smoke constituents generated from carbohydrates (simple sugars and cellulose). Formaldehyde formation from cellulose and starch is influenced by combustion and thermal degradation while from simple sugars it is predominantly formed by thermal degradation [10]. Acetaldehyde and formaldehyde are recognized as carcinogenic compounds. Generally, those compounds are more harmful inhaled than digested, because some metabolic pathways of detoxification are absent in the human respiratory system [53]. Formaldehyde is present in tobacco smoke in amounts up to 60 µg [10,53]. Baker et al. [10] reported that at temperatures under 500 °C, formaldehyde formation is avoided because sugars react with ammonium compounds and give other products, such as Amadori compounds. Moreover, an increase of formaldehyde content can contribute to the addictive potential of tobacco products.

Reaction of acetaldehydes with amino acids can result in the formation of Harman compounds. Harman compounds can inhibit enzyme monoamine oxidase (MAO). MAO is an enzyme that decomposes neurotransmitters included in drug addiction, such as dopamine and noradrenaline. Harman can be bound to neurotransmitter receptors or synergistically work with nicotine.

**Figure 3:**
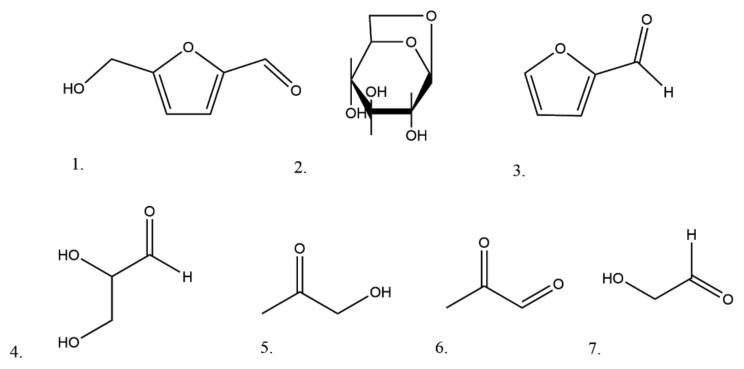
The chemical structures of some typical degradation compounds from tobacco carbohydrates (1. HMF, 2. Levoglucosan, 3. Furfural, 4. Glyceraldehyde, 5. Hydroxyacetone, 6. Pyruvic aldehyde and 7. Hydroxyacetaldehyde).

Other pyrolysis products from reducing sugars (glucose, fructose) and sucrose are furans, including furfural and 5-HMF, Hhydroxyacetone and levoglucosan (Figure 3). In research published by Jokić et al. [56], in the treatment of tobacco and tobacco waste with subcritical water at high temperatures (150, 200 and 250 °C), an increase of temperatures resulted in an increased yield of furfural and 5-hydroxymethylfurfural and, at the same time, decreased the content of carbohydrates. Similar research performed by Martin et al. [57] resulted in high levels of HMF and furfural as a result of the dehydration of pentoses and hexoses in tobacco stem material.

Mechanisms of formation of harmful compounds in tobacco smoke are complex and many are not known, and toxic effects of smoke depend not only on one single compound, but rather on combinations of different chemicals. Therefore, reducing levels of only one compound, such as formaldehyde, may not have an expected results for human health [10].

## 5. Tobacco Carbohydrates as Bioactive Compounds

For a long time, carbohydrates were considered only as primary plant metabolites but in recent times they have started to be considered as secondary metabolites (bioactive compounds) as well. Bioactive compounds are widely found in plants and have been used for the prevention of different illnesses and in the treatment of a wide range of diseases. Plants produce those compound as defense from biotic and abiotic stresses [58,59]. In 2013, Xu et al. reported for the first time that tobacco carbohydrates possess antioxidant activity [60]. They found that in tobacco leaf extracts, carbohydrate content correlated with antioxidant activity. The proposed mechanism suggested that COOH, -C- and C=O groups have the potential to reduce free radicals by donating electrons. By the proposed mechanism, more stable forms are formed or involved in further reactions with free radicals. Liu et al. [61] showed that in tobacco extracts, the polysaccharide content increased radical scavenging activity, showing the antioxidant potential of tobacco carbohydrates. They attributed radical scavenging activity to monosaccharides content, because extracts with higher content of monosaccharides expressed higher antioxidant activity.

Sugar esters are compounds that have been shown to possess many biological activities such as insecticidal, antifungal, and antibacterial properties [62]. Tobacco sugar esters provide pest resistance and could be considered as potential green pesticides. They are derived by acyl moieties esterified to the hydroxyl groups of sugars (sucrose or glucose). They are produced by glandular trichomes on the leaf surface of the *Solanaceace* family and are recognized as the main flavor precursors in *Oriental* tobacco [63,64]. Puterka et al. [65] claimed that sugar esters had higher insecticidal and miticidal properties than insecticidal soap. Generally, carbohydrate-based bioactive compounds are characterized with very complex chemical structure. That makes research on them very slow, but in the future their high bioactive potential and contribution in many physiological and pathological functions of organisms probably will be investigated more deeply [66].

## 6. Conclusions

For better understanding of tobacco carbohydrates and their influence on tobacco smoke chemistry, aroma properties and potential toxicity, it is crucial to understand changes in tobacco carbohydrates during processing from tobacco green leaf, through the formation of tobacco commercial blends to tobacco smoke. Also, the addition of carbohydrates during the leaf process should be taken into account and considered both together and apart from naturally present tobacco carbohydrates. This paper has highlighted the importance of carbohydrates, with particular emphasis on sugars. Finally, a number of potential limitations needs to be considered. Firstly, some of the published papers related to tobacco carbohydrates are performed by the tobacco industry, and obtained information should be taken cautiously. Secondly, even though there are many published papers about the relationship between carbohydrate contents and volatile compounds of tobacco, pathways from carbohydrates to aroma compounds are still not fully known. Further research should consider carbohydrates, including cellulose and pectin (not only simple sugars), in the formation of desirable tobacco aroma.

## Figures and Tables

**Table 1:** Chemical composition of three the most important varieties of tobacco.

(%)	TOBACCO VARIETY		
	*Burley*	*Virginia*	*Oriental*
Nicotine	2.07	2.26	1.18
Sugars	1.2	11.1	10.7
Proteins	10.1	7.8	7.8
Chlorine	0.51	0.53	0.84
Crude Ashes	18.4	12.8	16.4

## References

[1] Tayoub G., Sulaiman H., Alorfi M. (2015). Determination of nicotine levels in the leaves of some *Nicotiana tabacum* varieties cultivated in Syria. Herba Pol..

[2] Banožić M., Banjari I., Jakovljević M., Šubarić D., Tomas S., Babić J., Jokić S. (2019). Optimization of Ultrasound-Assisted Extraction of Some Bioactive Compounds from Tobacco Waste. Molecules.

[3] Goubet I., Le Quere J.-L., Voilley A.J. (1998). Retention of Aroma Compounds by Carbohydrates: Influence of Their Physicochemical Characteristics and of Their Physical State: A Review. J. Agric. Food Chem..

[4] Hall M.B.M., Merten S.D.R. (2017). 100-Year Review: Carbohydrates—Characterization, digestion, and utilization. J. Dairy Sci..

[5] Feng J.-W., Zheng S., Maciel G.E. (2004). EPR Investigations of Charring and Char/Air Interaction of Cellulose, Pectin, and Tobacco. Energ. Fuel..

[6] Roemer E., Schorp M.K., Piadé J.-J., Seeman J.I., Leyden D.E., Haussmann H.J. (2012). Scientific assessment of the use of sugars as cigarette tobacco ingredients: A review of published and other publicly available studies. Crit. Rev. Toxicol..

[7] Xi Y.X., Song J.Z., Yang J., Li F., Cai X.J., Wang X.M., Wei C.Y. (2011). Analysis of flavor precursors and degradation products content in flue-cured tobacco of different color and maturity. Acta Tab. Sin..

[8] Zhu W.K., Wang Y., Chen L.Y., Wang Z.G., Li B., Wang B. (2016). Effect of two-stage dehydration on retention of characteristic flavor components of flue-cured tobacco in rotary dryer. Dry. Technol..

[9] Mitsui K., David F., Dumont E., Ochiai N., Tamura H., Sandra P. (2015). LC fractionation followed by pyrolysis GC–MS for the in-depth study of aroma compounds formed during tobacco combustion. J. Anal. Appl. Pyrolysis.

[10] Baker R.R. (2006). The generation of formaldehyde in cigarettes—Overview and recent experiments. Food Chem. Toxicol..

[11] Weeks W.W. (1985). Chemistry of tobacco constituents influences flavor and aroma. Rec. Adv. Tob. Sci..

[12] Schaller J.P., Pijnenburg J.P.M., Ajithkumar A., Tricker A.R. (2016). Evaluation of the Tobacco Heating System 2.2. Part 3: Influence of the tobacco blend on the formation of harmful and potentially harmful constituents of the Tobacco Heating System 2.2 aerosol. Regul. Toxicol. Pharmacol..

[13] Ali H., Pätzold R., Brückner H. (2006). Determination of L- and D-amino acids in smokeless tobacco products and tobacco. Food Chem..

[14] Leffingwell J.C. (1976). Nitrogen components of leaf and their relationship to smoking quality and aroma. Rec. Adv. Tob. Sci..

[15] Baker R.R., Coburn S., Liu C., Tetteh J. (2005). Pyrolysis of saccharide tobacco ingredients: A TGA-FTIR investigation. J. Anal. Appl. Pyrolysis.

[16] Clarke M.B., Bezabeh D.Z., Howard C.T. (2006). Determination of Carbohydrates in Tobacco Products by Liquid Chromatography-Mass Spectrometry/Mass Spectrometry: A Comparison with Ion Chromatography and Application to Product Discrimination. J. Agric. Food Chem..

[17] Geiss O., Kotzias D. (2007). Tobacco, Cigarettes and Cigarette Smoke an Overview. Institute for Health and Consumer Protection.

[18] Adam T., Ferge T., Mitschke S., Streibel T., Baker R.R., Zimmermann R. (2004). Discrimination of three tobacco types (Burley, Virginia and Oriental) by pyrolysis single-photon ionisation? time-of-flight mass spectrometry and advanced statistical methods. Anal. Bioanal. Chem..

[19] Leffingwell J.C., Davis D.L., Mark T.N. (1999). Leaf Chemistry BA Basic Chemical Constituents of Tobacco Leaf and Differences among Tobacco Types Reprinted from Tobacco: Production, Chemistry, And Technology.

[20] Talhout R., Opperhuizen A., van Amsterdam J.G.C. (2006). Sugars as tobacco ingredient: Effects on mainstream smoke composition. Food Chem. Toxicol..

[21] Wu M., Wu X.L. (1996). A study of burley material. Tob. Sci. Technol..

[22] Li P., Wu M., Xie J. (2003). Changes in Levels of Amino Acids and Basic Components in Burley Tobacco Produced by Roasting. Contrib. Tob. Res..

[23] Cai K., Hu D., Lei B., Zhao H., Pan W., Song B. (2015). Determination of carbohydrates in tobacco by pressurized liquid extraction combined with a novel ultrasound-assisted dispersive liquid–liquid microextraction method. Anal. Chim. Acta.

[24] Nagai A., Yamamoto T., Wariishi H. (2012). Identification of Fructo- and Malto-oligosaccharides in Cured Tobacco Leaves (*Nicotiana tabacum*). J. Agric. Food Chem..

[25] Zook C.M., Patel P.M., LaCourse W.R., Ralapati S. (1996). Characterization of Tobacco Products by High-Performance Anion Exchange Chromatography−Pulsed Amperometric Detection. J. Agric. Food Chem..

[26] K Department of Health (2003). Permitted Additives to Tobacco Products in the United Kingdom.

[27] EU Tobacco Product Directive TPD (2014). Tobacco Product Directive 2014/40/EU of the European Parliament and of the Council.

[28] Friedmann M. (1996). Food browning and its prevention: An overview. J. Agric. Food Chem..

[29] Pilon-Smits E.A.H., Ebskamp M.J.M., Paul M.J., Jeuken M.J.W., Weisbeek P.J., Smeekens S.C.M. (1995). Improved performance of transgenic fructan-accumulating tobacco under drought stress. Plant Physiol..

[30] Song Z., Li T., Gong C. (2009). A review on starch changes in tobacco leaves during flue-curing. Front. Agric. China.

[31] Ridley B.L., O’Neill M.A., Mohnen D. (2001). Pectins: Structure, biosynthesis, and oligogalacturonide-related signaling. Phytochemistry.

[32] Lara-Espinoza C., Carvajal-Millán E., Balandrán-Quintana R., López-Franco Y., Rascón-Chu A. (2018). Pectin and Pectin-Based Composite Materials: Beyond Food Texture. Molecules.

[33] Zhang M., Zeng G., Pan Y., Qi N. (2018). Difference research of pectins extracted from tobacco waste by heat reflux extraction and microwave-assisted extraction. Biocatal. Agric. Biotechnol..

[34] Zhu X., Liu B., Zheng S., Gao Y. (2014). Quantitative and structure analysis of pectin in tobacco by 13C CP/MAS NMR spectroscopy. Anal. Methods.

[35] Baliga V., Sharma R., Miser D., McGrath T., Hajaligol M. (2003). Physical characterization of pyrolyzed tobacco and tobacco components. J. Anal. Appl. Pyrolysis.

[36] Liu B., Li Y.-M., Wu S.-B., Li Y.-H., Deng S.-S., Xia Z.-L. (2012). Pyrolysis Characteristic of Tobacco Stem Studied by Py-GC/MS, TG-FTIR, and TG-MS. BioResources.

[37] Tso T.C., Beltsville M.D., Davis D.L., Nielsen M.T., Ideals Inc. (1990). Alkaloids in Production, Physiology and Biochemistry of Tobacco Plant. Tobacco-Production, Chemistry and Technology.

[38] Tuzzin G., Godinho M., Dettmer A., Zattera A.J. (2016). Nanofibrillated cellulose from tobacco industry wastes. Carbohydr. Polym..

[39] Lu Q., Yang X., Dong C., Zhang Z., Zhang X., Zhu X. (2011). Influence of pyrolysis temperature and time on the cellulose fast pyrolysis products: Analytical Py-GC/MS study. J. Anal. Appl. Pyrolysis.

[40] Torikaiu K., Uwano Y., Nakamori T., Tarora W., Takahashi H. (2005). Study on tobacco components involved in the pyrolytic generation of selected smoke constituents. Food Chem. Toxicol..

[41] Sanders E.B., Goldsmith A.I., Seeman J.I. (2002). A model that distinguishes the pyrolysis of D-glucose, D-fructose, and sucrose from that of cellulose. Application to the understanding of cigarette smoke formation. J. Anal. Appl. Pyrolysis.

[42] Yamaguchi N., Suzuki S., Makino A. (2013). Starch degradation by alpha-amylase in tobacco leaves during the curing process. Soil Sci. Plant Nutr..

[43] Abubakar Y., Young J.H., Johnson W.H., Weeks W.W. (2000). Changes in moisture and chemical composition of flue-cured tobacco during curing. Tob. Sci..

[44] Zilkey B.F., Court W.A., Binns M.R., Basrur P.K. (1982). Chemical Studies on Canadian Tobacco and Tobacco Smok. 1. Tobacco, Tobacco Sheet, and Cigarette Smoke Chemical Analysis on Various Treatments of Bright and Burley Tobacco. Tob. Int..

[45] Pang T., Yuan Z., Dai Y., Wang C., Yang J., Peng L., Xu G. (2007). Identification and determination of glycosides in tobacco leaves by liquid chromatography with atmospheric pressure chemical ionization tandem mass spectrometry. J. Sep. Sci..

[46] Cerny C. (2008). The Aroma Side of the Maillard Reaction. Ann. N. Y. Acad. Sci..

[47] Hofmann T., Schieberle P. (1998). Identification of key aroma compounds generated from cysteine and carbohydrates under roasting conditions. Zeitschrift For Lebensmitteluntersuchung und -Forschung A.

[48] Liu L., Wang X., Wang S., Liu S., Jia Y., Qin Y., Liu H. (2017). Simultaneous quantification of ten Amadori compounds in tobacco using liquid chromatography with tandem mass spectrometry. J. Sep. Sci..

[49] Jia C., Xiu L., Mou D. (2015). Simultaneous determination of six kinds of Amadori 305 compounds in tobaccos by LC-MS/MS. J. Chin. Mass. Spectr. Soc..

[50] Zhou W., Wang J., Wu D. (2014). Determination of important flavour precursor compounds 303 (Amadori compounds) in cigarettes by LC-MS/MS. J. Anal. Chem..

[51] García-Moreno M.I., Benito J.M., Mellet C.O., Fernández J.M.G. (2008). Chemical and Enzymatic Approaches to Carbohydrate-Derived Spiroketals: Di-D-Fructose Dianhydrides (DFAs). Molecules.

[52] Tomasik P. (1989). The thermal decomposition of carbohydrates. Part I. The decomposition of mono-, di-, and oligo-saccharides. Adv. Carbohydr. Chem. Biochem..

[53] Shen D.K., Gu S. (2009). The mechanism for thermal decomposition of cellulose and its main products. Bioresourc. Technol..

[54] Husain Z., Ansari K.B., Chatake V.S., Urunkar Y., Pandit A.B., Joshi J.B. (2019). Valorisation of biomass pellets to renewable fuel and chemicals using pyrolysis: Characterisation of pyrolysis products and its application. Ind. Chem. Eng..

[55] Van Nierop L.E., Talhout R. (2016). Sugar as Tobacco Additive Tastes “Bitter”. J. Addict. Res. Ther..

[56] Jokić S., Gagić T., Knez Ž., Banožić M., Škerget M. (2019). Separation of active compounds from tobacco waste using subcritical water extraction. J. Supercrit. Fluids.

[57] Martin C., Fernandez T., Garcia R., Carrillo E., Marcet M., Galbe M., Jönsson L.J. (2002). Preparation of hydrolysates from tobacco stalks and ethanolic fermentation by *Saccharomyces cerevisiae*. World J. Microbiol. Biotechnol..

[58] Azmir J., Zaidula S.M., Rahmana M.M., Sharif K.M., Mohamed A., Sahena F., Jahurul M.H.A., Ghafoor K., Norulaini N.A.N., Omar A.K.M. (2013). Techniques for extraction of bioactive compounds from plant materials: A review. J. Food Eng..

[59] Cvjetko Bubalo M., Vidović S., Radojčić Redovniković I., Jokić S. (2015). Green solvents for green technologies. J. Chem. Technol. Biotechnol..

[60] Xu C.P., Xiao Y., Mao D.B. (2013). Antioxidant activities of polysaccharide fractions isolated from burley tobacco flowers. Croat. J. Food Sci. Technol..

[61] Liu S., He P., Tian Z., Li X., Xu C. (2015). Ultrasound-Assisted Extraction and Characterization of Polysaccharide From Maryland Tobacco Leaves. J. Chil. Chem. Soc..

[62] Popova V., Ivanova T., Stoyanova A., Nikolova V., Hristeva T., Gochev V., Yonchev Y., Nikolov N., Zheljazkov V.D. (2020). Terpenoids in the Essential Oil and Concentrated Aromatic Products Obtained from *Nicotiana glutinosa* L. Leaves. Molecules.

[63] Kroumova A.B.M., Zaitlin D., Wagner G.J. (2016). Natural variability in acyl moieties of sugar esters produced by certain tobacco and other *Solanaceae* species. Phytochemistry.

[64] Huang Z., Bi Y.J., Sha Y.-F., Xie W.Y., Wu D., Liu B.-Z. (2018). Separation and Analysis of Sucrose Esters in Tobacco by Online Liquid Chromatography–Gas Chromatography/Mass Spectrometry. Anal. Sci..

[65] Puterka G.J., Farone W., Palmer T., Barrington A. (2003). Structure-Function Relationships Affecting the Insecticidal and Miticidal Activity of Sugar Esters. J. Econ. Entomol..

[66] Cipolla L., Peri F. (2011). Carbohydrate-based bioactive compounds for medicinal chemistry applications. Mini Rev. Med. Chem..

